# The Prevalence of Virulence Genes and Multidrug Resistance in Thermophilic *Campylobacter* Spp. Isolated from Dogs

**DOI:** 10.1515/biol-2019-0077

**Published:** 2019-12-31

**Authors:** Marek Selwet

**Affiliations:** 1 Department of General and Environmental Microbiology, Poznań University of Life Sciences, ul. Szydłowska 28, 60-637 Poznań, Poland

**Keywords:** campylobacteriosis, antibiotics, toxin CDT, virulence genes

## Abstract

The aim of the study was to determine the role of dogs as a potential reservoir of *Campylobacter* spp. At the next stage of the research the frequency of occurrence of selected virulence genes, i.e. *cadF*, *flaA* and *iam* as well as genes responsible for the formation the cytolethal distending toxin (CDT), i.e. *cdtA*, *cdtB* and *cdtC* was determined. The isolates obtained in the research were tested for their resistance to selected antibiotics: ciprofloxacin (CIP), enrofloxacin (EF), erythromycin (E) and tetracycline (TE). *Campylobacter* spp. was found in 63 (12.6%) out of a total number of 500 isolates. 61 (12.2%) isolates were identified as *C. jejuni*. The number of *C. jejuni* isolates found in the younger animals was smaller (*p* <0.05) than in the older ones. The frequency of occurrence of virulence genes and the genes responsible for the formation of CDT was significantly (*p* <0.05) higher in the older dogs. A comparison of the effect of antibiotics showed that the isolates obtained from both age groups exhibited low resistance to erythrosine (13.5% in the group aged under 1 year and 8.6% in the group aged over 1 year). Both groups exhibited the highest resistance to ciprofloxacin and enrofloxacin.

## 1 Introduction

Thermotolerant bacteria of the *Campylobacter* genus (mainly *Campylobacter jejuni* and *Campylobacter coli*) are part of the natural intestinal flora in mammals and birds. They can also be found in water and soil contaminated with animal faeces [1]. As these bacteria are so common, animal products, especially red meat and poultry, are at risk of contamination. Close contact with a sick animal and lack of hygiene may cause campylobacteriosis in humans and become a serious epidemiological problem in the 21^st^ century [2, 3]. The incidence of this disease in humans has been increased in Europe since 2005 [4]. In 2015 the overall incidence of infections in the EU was 65.5 cases per 100.000 inhabitants [5]. 20-40% of human infections were caused by contact with poultry or poultry meat [6]. The most common clinical symptoms are diarrhea, fever and abdominal pain. The infection usually disappears spontaneously, although there may be complications such as arthritis [7]. According to the latest EFSA report [8], each year about 200.000 cases of campylobacteriosis infections diagnosed in humans were caused by the consumption of contaminated food. The actual number of infections is estimated at 9 million per annum. According to the EFSA estimates, the annual costs generated by the incidence of campylobacteriosis in public healthcare in the EU amount to 2.4 billion Euros (€). Due to the fact that *Campylobacter* spp. isolates from humans are increasingly often resistant to quinolone chemotherapeutics [9], aminoglycosides and macrolide antibiotics, they pose a threat to public health [10]. The development of resistance is often associated with spontaneous point mutations of the genes encoding enzymes synthesised by *Campylobacter* spp. [11]. The bacteria were proved to have various pathogenic properties resulting from high genetic diversity of their strains. The pathogenicity of bacteria is caused by the genes that determine their motility, adhesion, invasiveness and synthesis of a cytolethal distending toxin (CDT), which is encoded by three genes – *cdtA*, *cdtB* and *cdtC*. This cytotoxin causes cell cycle arrest at the G2/M phase [12]. Other virulence genes are: *fla*, *cad*, *rac*, *vir*, *cia*, *pld*, *iam*. The *fla* genes (*flaA* and *flaB*) are responsible for bacterial motility. They encode flagellin – the ciliary

protein which enables *Campylobacter* spp. cells to move. The *cadF* gene encodes the fibronectin binding protein of enterocytes, which participate in adherence. Many researchers believe that this gene, which is necessary to induce symptoms of campylobacteriosis, is a conservative gene in *C. jejuni* and *C. coli*. The *vir* gene, which can be found in the *Campylobacter* spp. plasmid (the plasmid is not always present), also encodes proteins responsible for pathogenicity. The *iam* sequence, which is responsible for adhesion and invasiveness, is found in *C. coli* more often than in *C. jejuni* [13]. In consequence, it may disorder intestinal absorption.

The aim of the study was to determine the frequency of occurrence of *Campylobacter* bacteria in dogs, to identify species within the *Campylobacter* genus, selected virulence genes in isolated strains and genes determining the occurrence of CDT, and to determine resistance to selected antibiotics.

## 2 Materials and methods

### 2.1 Sampling

Swab samples (swabs with transport medium, Stuart swabs. Sterile, product No. 300295, Deltalab, Spain) collected from the rectum of 500 dogs (*n* = 250 dogs younger than 1 year and *n* = 250 dogs older than 1 year) from shelters located in the Wielkopolska region were used as material for the tests. No dog showed gastrointestinal symptoms. The samples were collected on three consecutive days and transported to a laboratory within 6 hours while stored at 4°C.

**Ethical approval**: The research related to animals use has been complied with all the relevant national regulations and institutional policies for the care and use of animals.

### 2.2 Isolation and identification of Campylobacter spp. from swab samples (PN EN ISO10272 under modification)

Swab samples were initially placed in 3 cm^3^ of liquid selective medium Preston Broth No. 2 (product No. CM067, Lysed Horse Blood product No. SR0048, Preston Campylobacter Selective Supplement product No. SR0117 and Campylobacter Growth Supplement product No. SR0232, Oxoid). Next, they were incubated in a CampyGen anaerostat (product No. CN0025, Oxoid) at 41.5 °C/24 h (5% O_2_, 10% CO_2_, 85% N_2_). After incubation the bacterial cultures were centrifuged (4.000 x g/15 min), the supernatant was poured and the sediment was added to 1 cm^3^ of Karmali agar. 200 μL of the suspension was spread with a spreader on a selective Karmali agar (product No. CM0935, Oxoid) and incubated in an anaerostat at 41.5 °C/48 h. An API Campy biochemical test, product No. 20800 (Biomérieux) was used to confirm the presence of *Campylobacter* spp. The identity of the colonies grown on the agar was determined by observation of their morphological features and motility (Axio Imager.A2 microscope, Zeiss). The following tests were carried out: oxidase (OXI detection strip, product No. 2001, Diagnostics Inc., Slovak Republic), catalase (API ID colour catalase, product No. 55561, Biomérieux) and the hydrolysis capacity of hippurate and indoxyl acetate (HIP , product No. 2006 and HIP reagent, product No. 3006; INDOXYL, product No. 2007, Diagnostics Inc., Slovak Republic). A selective mCCDA medium (Oxoid) was used for a confirmatory test, where colonies grown after incubation at 41.5 °C/48 h were counted. *Campylobacter* spp. was also distinguished from other gram-negative bacteria by means of an O.B.I.S. Campy test (product No. ID0800M, Oxoid). Real-time PCR (Biorad) with the BAX System Real-Time PCR Assay for Campylobacter (product No. D12683449 KIT2018, Hygiena) was used for species identification.

### 2.3 Identification of genes determining CDT occurrence by means of multiplex PCR

The primers shown in Table 1 were used to identify the *cdtA*, *cdtB*, and *cdtC* genes. A 25μL reaction mixture was used. It was composed of: 2.5 μL 10 x PCR buffer, 6 x 1 μL primers (5 μM), 1 μL dNTP (2.5 mM), 0.2μL *Taq* polymerase, 1.8 μL DNA, 13.5 μL water. The thermal conditions of the reaction were as follows: initial denaturation at 94 °C/2 min. It was followed by 30 cycles, each of which consisted of: denaturation (94 °C/0.5 min), primer attachment (50 °C/0.5 min) and extension (72 °C/1 min and 72 °C/5 min). The resulting products were analysed by means of electrophoresis in 1.5% agarose gel.

**Table 1:** Sequences of primers used to detect genes *cdtA*, *cdtB*, *cdtC*.

Primer	Sequences 5^’^ →3^’^	Product size (bp)	References
*cdtA*-F	CTA TTA CTC CTA TTA CCC CAC C	422	[14]
*cdtA*-R	AAT TTG AAC CGC TGT ATT GCT C		
*cdtB*-F	AGG AAC TTT ACC AAG AAC AGC C	531	
*cdtB*-R	GGT GGA GTA TAG GTT TGT TGT C		
*cdtC*-F	ACT CCT ACT GGA GAT TTG AAA G	339	
*cdtC*-R	CAC AGC TGA AGT TGT TGT TGG C		

### 2.4 Identification of virulence genes

*cadF*, *flaA*, and *iam* genes were identified by means of the primers shown in Table 2. The reaction was carried out in a total volume of 25μL. The mixture was composed of: 2.5 μL 10 x PCR buffer, 2.5 μL MgCl_2_ (25 mM), 1 μL dNTP (2.5 mM), 1 μL primers (5 μM), 0.2 μL (1U ) U *Taq*

**Table 2:** Sequences of primers used to detect genes *cadF, flaA, iam*.

Primer	Sequences 5^’^ →3^’^	Product size (bp)	References
*cad F*-F	TGGAGGGTAATTTAGATATTG	400	[15]
*cad F*-R	CTAATACCTAAAGTTGAAAC		
*fla A*-F	GGATTTCGTATTAACACAAATGGTGC	1728	[16]
*fla A*-R	CTGTAGTAATCTTAAAACATTTTG		
*iam*-F	GCGCAAAATATTATCACCC	518	[17]
*iam*-R	TTCACGACTACTATGCGG		

DNA polymerase (Promega Corporation), 2 μL DNA, 15, 5 μL water. The thermal conditions of the reaction were as follows: initial denaturation (94 °C/1 min). It was followed by 30 cycles, each of which consisted of: denaturation (94 °C/0.5 min), primer attachment (45 °C/1 min), extension (72 °C/2 min and 72 °C/5 min). The resulting products were analysed by means of electrophoresis in 1.5% agarose gel.

### 2.5 Assessment of sensitivity to antibiotics

The isolated *Campylobacter* bacterial strains were tested for sensitivity to antibiotics by means of the Kirby-Bauer disc diffusion test and E-test. The method was compatible with the EUCAST [18] Disc Diffusion Method for Antimicrobial Susceptibility Testing and Clinical and Laboratory Standards Institute (CLSI). Bacterial colonies were grown on lysogeny broth (LB) (product No. L3522, Merck). After 24 h incubation at 37 °C the culture was diluted to a density of 0.5 on the McFarland scale. The suspension was diluted at a ratio of 1:10 again and a surface culture was prepared on Mueller-Hinton Agar (product No. 70191, Merck) with 20 mg/L ß-NAD (product No. 10127981001, Merck). The discs were then coated with antibiotics: ciprofloxacin (CIP) 5 μg, erythromycin (E) 15 μg, tetracycline (TE) 30 μg and enfofloxacin (EF) 5 μg and incubated at 41 ± 1 °C/24 h. An analogous procedure was applied in the E test, where strips coated with antibiotics were used to determine the MIC on the scale of a NEMA 88 device (product No. 559804, Biomérieux).

*C. jejuni* ATCC 33291 and *C. coli* ATCC 33559 (DSMZ Germany) were used as reference strains in the study.

### 2.6 Statistical analysis

The counts of microorganisms measured in the investigations were analysed statistically using the GLM procedure of the SAS program. The significance of differences was verified with Duncan’s test (α=0.05).

## 3 Results

### 3.1 Isolation and identification of *Campylo-bacter* spp

*Campylobacter* spp. was found in 63 (12.6%) out of the total number of 500 isolates. 61 (12.2%) isolates were identified as *C. jejuni* (Table 3). There were 2 isolates (0.4%) that should not pose a threat to human health, so they were not further differentiated. There were no *C. coli* or *C. lari* strains detected. The total number of positive isolates found in the dogs aged under 1 year was lower (*n* = 27; 10.8%) than the number of positive isolates found in the dogs aged over 1 year (*n* = 36; 14.4%). The analysis of the species composition of the isolates (*n* = 61; 100%) showed that all of them belonged to *C. jejuni*. There were 26 (10.4%) *C. jejuni* isolates found in the dogs aged under 1 year. This number was lower than in the group of dogs aged over 1 year (*n* = 35, 14.0%). The differences were statistically significant (*p* <0.05).

**Table 3:** The prevalence of *Campylobacter* isolated from dogs.

	Age	*C. jejuni*		*C. coli*		*C. lari*		*C. species*		Negative		Positive		Total samples	
		*n*	%	*n*	%	*n*	%	*n*	%	*n*	%	*n*	%	*n*	%
Dogs	<1year	26a	10.4	0	0	0	0	1	0.4	223	89.2	27a	10.8	250	50
	>1 year	35b	14.0	0	0	0	0	1	0.4	214	85.6	36b	14.4	250	50
Total		61	12.2	0	0	0	0	2	0.4	437	87.4	63	12.6	500	100

a, b-means in columns designated with the same letters do not differ significantly at the level of *p*<0.05.

### 3.2 Identification of genes determining CDT occurrence

There were 3 dogs aged under 1 year (11.5%) and 15 dogs aged over 1 year (42.8%) with the *cdtA* gene. The *cdtB* gene was found in 2 dogs aged under 1 year (7.7%) and 17 dogs aged over 1 year (48.6%). The *cdtC* gene was found in 4 dogs aged under 1 year (15.3%) and 20 dogs aged over 1 year (57.1%). The differences were statistically significant (*p* <0.05). The frequency of occurrence of the genes determining CDT occurrence was higher in the animals aged over 1 year (Table 4).

**Table 4:** The number and percentages of virulence genes in *C. jejuni* isolated from dogs.

	Age	*cadF*	*flaA*	*iam*	*cdtA*	*cdtB*	*cdtC*
Dogs	<1 year (*n=*26)	16 (61.5)a	15 (57.7)a	9 (34.6)a	3 (11.5)a	2 (7.7)a	4 (15.3)a
	>1 year (*n=*35)	35 (100)b	35 (100)b	23 (65.7)b	15 (42.8)b	17 (48.6)b	20 (57.1)b
Total	*n=*61	51 (83.6)	50(82)	32 (52.4)	18 (29.5)	19 (31.1)	24 (39.3)

a, b-means in columns designated with the same letters do not differ significantly at the level of *p*<0.05.

### 3.3 Identification of virulence genes

There were 16 dogs aged under 1 year (61.5%) and 35 dogs aged over 1 year (100%) with the *cadF* gene. The *flaA* gene was found in 15 dogs aged under 1 year (57.7%) and 35 dogs aged over 1 year (100%). The *iam* gene was found in 9 dogs aged under 1 year (34.6%) and 23 dogs aged over 1 year (65.7%). The differences were statistically significant (*p* <0.05). The frequency of occurrence of the sequences of virulence genes was higher in the dogs aged over 1 year (Table 4).

### 3.4 Sensitivity to antibiotics (the percentage of resistant isolates is the average of two tests)

Tables 5-6 show the results of *Campylobacter jejuni* sensitivity (S) and resistance (R) to the antibiotics applied in the experiment. The E Test and disc diffusion test were two methods compared in the study. Enrofloxacin was the only antibiotic which resulted in statistically significant differences (*p* <0.05) between the methods, both in the group of dogs aged under 1 year and in the group of older animals. The isolates from the younger animals exhibited the highest resistance to ciprofloxacin (46%) and enrofloxacin (30.7%). The analysis of resistance of the isolates obtained from the older animals showed that they also exhibited high sensitivity to ciprofloxacin (50%) and enrofloxacin (22.9%) (this was observed only in the disc diffusion test). Both the isolates from the younger and older dogs exhibited low resistance to erythromycin (13.5% and 8.6%) and tetracycline (7.7% and 8.6%).

**Table 5:** Results of susceptibility testing of 26 isolates *C.jejuni* by disc diffusion and E test methods for four antibiotics (dogs <1 year).

Antimicrobial agents	E Test	Disc diffusion
Ciprofloxacin (5μg)	14S (*MIC≤1μg mL^-1^)	14S (≥21 mm)
	12R (MIC≥4μg mL^-1^)	12R (≤15 mm)
Enrofloxacin (5μg)	16S (MIC≤1μg mL^-1^)a	20S (≥23 mm)b
	10R (MIC≥2μg mL^-1^)a	6R (≤16 mm)b
Erythromycin (15μg)	22S (MIC≤1μg mL^-1^)	23S (≥23 mm)
	4R (MIC≥2μg mL^-1^)	3R (≤13 mm)
Tetracycline (30μg)	23S (MIC≤1μg mL^-1^)	25S (≥19 mm)
	3R (MIC≥2μg mL^-1^)	1R (≤14 mm)

* Minimum inhibitory concentration (MIC); S-susceptible, R-resistant; a, b-means in rows designated with the same letters do not differ significantly at the level of *p*<0.05.

**Table 6:** Results of susceptibility testing of 35 isolates *C.jejuni* by disc diffusion and E test methods for four antibiotics (dogs >1 year).

Antimicrobial agents	E Test	Disc diffusion
Ciprofloxacin (5μg)	17S (*MIC≤1μg mL^-1^)	18S (≥21 mm)
	18R (MIC≥4μg mL^-1^)	17R (≤15 mm)
Enrofloxacin (5μg)	34S (MIC≤1μg mL^-1^) a	20S (≥23 mm) b
	1R (MIC≥2μg mL^-1^) a	15R (≤16 mm) b
Erythromycin (15μg)	33S (MIC≤1μg mL^-1^)	31S (≥23 mm)
	2R (MIC≥2μg mL^-1^)	4R (≤13 mm)
Tetracycline (30μg)	32S (MIC≤1μg mL^-1^)	33S (≥19 mm)
	3R (MIC≥2μg mL^-1^)	3R (≤14 mm)

* Minimum inhibitory concentration (MIC); S-susceptible, R-resistant; a, b-means in rows designated with the same letters do not differ significantly at the level of *p*<0.05.

## 4 Discussion

Initially, biochemical tests were used to detect species such as *C. coli* and *C. jejuni* in research on *Campylobacter* spp. Particular attention was paid to the ability of *C. jejuni* to hydrolyse sodium hippurate. Since the description of hippurate-negative *C. jejuni* strains it has seemed more reliable to use the PCR method and the Real-Time PCR system with complementary primers [19]. Our study revealed a low incidence of *Campylobacter* spp. in the group of dogs (*n* = 500), i.e. 12.6%. This result stood in opposition to our earlier studies [1, 20] and research conducted by other authors [5, 21]. Chaban et al. [22] analysed a group (*n* = 70) of healthy dogs and found bacteria of the *Campylobacter* genus in 58% of the samples. Likewise, the authors did not find *C. coli* or *C. lari*, whereas *C. jejuni* was detected in 7% (*n* = 5) of the positive samples. Differences in the results of studies conducted by various authors may have been caused by many factors, e.g. the sampling method, the culturing conditions in a laboratory (in vitro) or high species diversity within the *Campylobacter* genus [23]. Other authors have suggested that the age of animals may affect the population of *Campylobacter* spp. [24]. Hald et al. [25] noticed a relation between the age of animals and the incidence of thermotolerant *Campylobacter* spp. The authors observed that the incidence of these bacteria increased from 60% in young animals (3 months old) to 100% in animals aged 12 months, but it then decreased to 67% in dogs aged 24 months.

The *C. jejuni* genome WCTC 11168, sequenced in 2000, revealed the presence of genes encoding a protein with potential toxic properties [19]. The strain contained *cdt* genes encoding the CDT protein (cytolethal distending toxin). These were two genes encoding proteins with haemolytic domains and one phospholipase gene. CDT was the first described genotoxin that damages the genetic material of sensitive eukaryotic cells by stopping the cell cycle and causing the death of cells as a consequence [26]. A CDT locus consists of three jointly constitutively transcribed open reading frames (ORFs): *cdtA*, *cdtB* and *cdtC*, which are usually located on the chromosome [27]. All three genes were detected in our study. Hyun-Ho et al. [28] recorded similar results in their study on dogs. It is noteworthy that the frequency of occurrence of these genes in the dogs aged over 1 year was higher than in the younger animals. The *cdtB* subunit is responsible for the nucleolytic effect. In our study the occurrence of this gene in older dogs was higher than in the younger animals, i.e. 48.6% vs. 7.7%. Other authors have observed higher values. Andrzejewska et al. [29] researched a group of dogs and found that the percentage of animals with *cdtB* was as high as 72.7% or even 100% [30], whereas Findik et al. [31] noted 97.1%. The role of the *cdtA* and *cdtC* subunits should not be neglected either, because they may transport the *cdtB* subunit into the target cell (Krutkiewicz et al.) [19].

The pathogenesis of campylobacteriosis may be directly related with factors such as: motility, chemotaxis (mutants without these features do not colonise intestinal epithelial cells) as well as adhesion and invasiveness. Many genes and the effects of their expression are thought to be responsible for pathogenicity, e.g. *fla* – the motility gene, *cadF* – the adhesion gene and *iam* – the invasiveness gene [30, 32]. Our study showed a high percentage of the *cadF*, *flaA* genes and *iam* sequence in both groups of dogs. Rodrigues et al. [33] observed a high incidence of the *cadF* and *flaA* genes in dogs and noted that these genes may act as reporters in research on the mechanisms of *C. jejuni* pathogenicity.

The progressive increase in bacterial resistance is closely correlated with the misuse of chemotherapeutics. Apart from that, as a result of the widespread application of antibiotics to animals, often without medical indications, bacteria become selected towards antibiotic resistance. In consequence, they develop multi-drug resistance [34]. *Campylobacter* spp. strains isolated from animals and food exhibited the highest resistance to ciprofloxacin and tetracycline [8]. The main problem with *Campylobacter* spp. is the resistance of these bacteria to aminoglycosides (gentamicin), macrolides (erythromycin) and quinolones (ciprofloxacin), which are commonly used to treat campylobacteriosis [4]. Research conducted in Poland between 2003 and 2006 showed that all strains isolated from humans were sensitive to erythromycin. On the other hand, the percentage of clinical strains of *Campylobacter jejuni* which were resistant to ciprofloxacin was very high, i.e. 58% [35]. Research conducted in the US showed that 99.5% of *Campylobacter* spp. strains isolated from poultry were resistant to one or more antibiotics. 99.5% of *C. jejuni* strains and 96.3% of *C. coli* strains were resistant to tetracycline. Research conducted in Slovenia also showed a high percentage of *C. jejuni* isolates resistant to enrofloxacin (58.2%). The strains were less resistant to erythromycin (14.5%) and tetracycline (12.7%) [36]. These results are in line with our research findings. Maćkiw et al. [37] observed the highest resistance to ciprofloxacin (97.9%), and lower resistance to tetracycline (64.3%) and erythromycin (9.1%). The authors noted that 7% of 143 resistant isolates exhibited resistance to 3 unrelated antibiotics. Research conducted on dogs in India [36] revealed that all the isolates were resistant to tetracycline (100%), and most of them were resistant to ciprofloxacin and enrofloxacin (97.3%). The values recorded by these authors were higher than the values noted in our study.

## 5 Conclusions

To sum up, the dogs were identified as asymptomatic carriers of *Campylobacter* spp. and were found to be vectors carrying and transmitting *Campylobacter* infections to humans. It is necessary to conduct further research on these bacteria as risk factors to better understand the epidemiology of *Campylobacter* spp. and to emphasise the dangers of the unreasonable use of antibiotics in both human and animal therapy.
